# *Lantana camara* leaf extract ameliorates memory deficit and the neuroinflammation associated with scopolamine-induced Alzheimer’s-like cognitive impairment in zebrafish and mice

**DOI:** 10.1080/13880209.2023.2209130

**Published:** 2023-05-22

**Authors:** Veronica Amoah, Paul Atawuchugi, Yakubu Jibira, Augustine Tandoh, Paul Poku Sampene Ossei, George Sam, George Ainooson

**Affiliations:** a Department of Pharmacology, Kwame Nkrumah University of Science and Technology (KNUST), Kumasi, Ghana; b Department of Pharmacology and Toxicology, University of Development Studies, Tamale, Ghana; c Department of Pharmacology and Toxicology, University of Health and Allied Sciences, Ho, Ghana; d Department of Pathology, Kwame Nkrumah University of Science and Technology, Kumasi, Ghana; e Department of Herbal Medicine, Kwame Nkrumah University of Science and Technology, Kumasi, Ghana

**Keywords:** Alzheimer’s disease, qRT-PCR, T-maze, Y-maze, cytokines

## Abstract

**Context:**

*Lantana camara* Linn. (Verbenaceae) is used for improving memory in certain African societies.

**Objective:**

This study investigated the effect of prophylactic treatment with hydroethanolic leaf extract of *Lantana camara* (LCE) on short-term memory deficit and neuroinflammation induced with scopolamine in zebrafish and mice.

**Materials and methods:**

Zebrafish (AB strain) and mice (ICR) were given donepezil (0.65 mg/kg, oral) and LCE (10, 30, 100 mg/kg, oral) for 7, and 10 days, respectively, before induction of cognitive impairment with scopolamine immersion (200 µM) and intraperitoneal injection (2 mg/kg), respectively. Spatial short-term memory was assessed in zebrafish using both Y- and T-mazes, whereas Y-maze was used in mice. Mice hippocampal and cortical tissues were analyzed for mRNA expression of proinflammatory genes (IL-1β, IL-6, TNF-α, COX-2) using qRT-PCR.

**Results:**

In the zebrafish Y-maze, LCE (10 and 100 mg/kg) increased time spent in the novel arm by 55.89 ± 5.70%, and 68.21 ± 2.75%, respectively, but not at 30 mg/kg. In the zebrafish T-maze, there was an increase in time spent in the food-containing arm at 30 (44.23 ± 2.13) and 100 mg/kg (52.30 ± 1.94). In the mouse Y-maze, spontaneous alternation increased by 52.89 ± 4.98% at only 10 mg/kg. LCE (10, 30, 100 mg/kg) inhibited proinflammatory gene (IL-1β, IL-6, TNF-α, COX-2) mRNA expression, with the highest inhibitory effect on IL-6 in both the hippocampus (83.27 ± 2.49%; 100 mg/kg) and the cortex (98.74 ± 0.11%; 10 mg/kg).

**Discussion and conclusion:**

LCE ameliorated scopolamine-induced AD in both zebrafish and mice.

## Introduction

Alzheimer’s disease (AD) is a complex, multifactorial, progressive neurodegenerative disorder with loss of cognitive functions such as learning and memory as the defining feature (Mohs et al. 2000; Alzheimer’s Association 2021) and deficits in short-term memory as the earliest symptom (Götz et al. 2018). AD is said to be the most common form of dementia, accounting for 60-70% of cases as of 2021 (World Health Organization (WHO), 2021), and is predicted to reach epidemic proportions owing to longer life expectancy and the absence of an effective treatment (Götz et al. 2018).

AD development involves an interplay of several pathophysiological events that include amyloid beta (Aβ) plaques and neurofibrillary tangles (NFTs) formation, cholinergic system impairment, neuroinflammation, and oxidative stress. Aβ plaques and NFTs formation are the two classical pathological hallmarks (Huang and Mucke, 2012; Karthivashan et al. 2018).

Aβ plaques cause neuroinflammation *via* the activation of the resident immune cells of the brain, microglia, to cause aberrant expression of proinflammatory mediator genes (IL-1β, IL-6, TNF-α, and COX-2) (Lee et al. 2013; Liu et al. 2019). These genes promote AD pathologies *via* crosstalk between innate and adaptive immunities to drive neuronal cells toward apoptotic decline (Li et al. 2000; Dorey et al. 2014). They can induce the generation of Aβ plaques to promote neurotoxic effects in the brain (Blasko et al. 2004; Alasmari et al. 2018). Aβ plaques also promote caspase activation leading to the formation of NFTs (Zhang et al. 2021). NFTs are hyperphosphorylated tau proteins that accumulate in neurons and compromise axonal transport leading to loss of neuronal function and death of neurons (Alonso et al. 2008). Aβ plaque-induced loss of cholinergic neurons is a principal mechanism that results in memory loss and attention deficit (Ferreira-Vieira et al. 2016).

Cholinergic neurons synthesize and secrete the neurotransmitter acetylcholine which promotes learning and memory (Stancampiano et al. 1999). Thus, degeneration of cholinergic neurons results in decreased levels of acetylcholine resulting in cognitive impairment (Schliebs and Arendt 2011). Not surprisingly, the few drugs commonly available for AD, galantamine, donepezil, rivastigmine, and memantine, modulate the cholinergic system. Unfortunately, they have limited efficacy and are thus used only for symptomatic management (Alzheimer’s Association 2021) as they offer only minimal temporary improvements for about 1-3 years without influence on the disease progression (Sun et al. 2008). Also, Aducanumab, a monoclonal antibody that binds aggregated forms of Aβ plaques, that is currently under accelerated approval by the Food and Drugs Authority (FDA) of the USA, marks the first AD treatment to prevent disease progression (Ross et al. 2022; Sinha and Barocas 2022). However, this drug is not cost-effective (Ross et al. 2022; Sinha and Barocas 2022) as the cost outweighs any benefit that may be derived from its use. These and other challenges continue to drive the search for novel AD therapeutics that will be highly efficacious, safe, and cost-effective.

Natural products, especially medicinal plants, have been recommended as possible sources of lead molecules for novel therapeutics in AD (Roy 2018). The uses and pharmacological effects in the cholinergic pathway of a number of these plants such as *Ginkgo biloba* Linn. (Ginkgoaceae) and *Bacopa monnieri* Linn. (Scrophulariacae) (Saraf et al. 2011; Roy 2018) have already been described. *Lantana camara* Linn. (Verbenaceae), the subject of this investigation, is one such plant whose leaves are reportedly used in certain African societies for the treatment of inflammatory conditions (Adeniyi et al. 2018) and for improving memory (Muller-Ebeling and Ratsch 1989), but very little is known about its possible use in AD. It is therefore prudent under the current challenges to investigate such a plant for possible use in AD.

The scopolamine-induced Alzheimer’s-like cognitive impairment model is one of the simple and quick ways for studying the cognition-enhancing properties (Sarter et al. 2003; Klinkenberg and Blokland 2010) of new drugs (Zanandrea et al. 2018). This is because scopolamine mimics both the behavioral (cognitive impairment) and molecular (formation of neurotoxic Aβ plaques, ACh-deficiency, neuroinflammation) features of the disease (Chen and Yeong, 2020; Karthivashan et al. 2018). Mechanistically, scopolamine-induced Aβ plaques inhibit the synthesis of ACh by decreasing the activity of choline acetyltransferase (ChAT), an enzyme associated with the synthesis of ACh. Also, Aβ plaques inhibit the release of ACh and reduce choline uptake to cause ACh deficiency, manifesting as cognitive impairment similar to what occurs in human AD (Kar et al. 1998; Nunes-Tavares et al. 2012). This work determined the effect of prophylactic treatment with hydroethanolic leaf extract from *Lantana camara* (LCE) on short-term memory deficit and the associated neuroinflammation induced with scopolamine in zebrafish and mice.

## Materials and methods

### Drugs and chemicals

Scopolamine hydrobromide 99% (Xi an Sost Biotech Co. Ltd., China), donepezil hydrochloride (Cipla EU Ltd., UK), ethanol (NedStar, Amsterdam), propylene glycol (SKC Co. Ltd., South Korea) and ethyl 3-aminobenzoate methanesulfonate salt 98% (Sigma Aldrich, USA) were purchased.

### Animals

All experimental procedures used in this study were approved by the Animal Research Ethics Committee (AREC) of the Kwame Nkrumah University of Science and Technology (KNUST), Kumasi, Ghana [number KNUST 0013]. This ethical clearance covered the study.

### Zebrafish

Adult (4-month-old, 0.4–0.7 g) wild-type zebrafish (AB strain) of both sexes were obtained from the zebrafish facility of the Department of Pharmacology, Faculty of Pharmacy and Pharmaceutical Sciences, Kwame Nkrumah University of Science and Technology (KNUST), Kumasi, Ghana. Animals were kept in an Aqua Medic fish housing system (Bissendorf, Germany) fitted with fish tanks containing fish water [E3-medium; 0.29 g NaCl, 0.08 g MgSO_4_, 0.04 g CaCl_2_, and 0.01 g KCl per 1 L]. The fish were housed at a density of 5 animals per 3 L and maintained under a 12 h light**/**dark cycle. The temperature of fish water was generally kept between 26–28.5 °C and the pH was maintained at 6.8–7.5. Animals were fed three times daily with TetraMin Tropical Flakes (TetraGMBH, Herrenteich, Germany) at 3 h intervals. Experimental procedures were performed according to the European Union recommended guidelines for experiments with zebrafish [Directive 2010/63/EU of the European Parliament and of the Council of 22 September 2010 on the protection of animals used for scientific purposes (Text with EEA relevance)]. The feeding and swimming pattern of the fish were monitored throughout the experiment to determine suffering and ill health.

### Mice

Male ICR mice (25–30 g) were obtained from the Animal House of the Department of Pharmacology, KNUST. The animals were kept under standard environmental conditions of temperature (25–28 °C), relative humidity (60–70%), and normal cycles of 12 h light**/**dark. Mice had access to commercial food and clean water without restrictions. Handling of animals was performed under the National Institute of Health Guidelines (National Research Council (US) Guide for the Care and Use of Laboratory Animals Eighth Edition 2011). The grooming, eating, drinking, and interacting behavior among mice were monitored throughout the experiment to determine suffering and ill health.

### Plant material and extraction procedure

Fresh leaves of *Lantana camara* were collected on March 2020 from Mampong in the Ashanti Region of Ghana and authenticated by Dr. George Sam at the herbarium of the Department of Herbal Medicine, KNUST. A voucher specimen (KNUST/HM1/2021/L019) was kept at the herbarium. The leaves of the plant were washed thoroughly, dried in shade, and powdered coarsely. Approximately 780 g of the powdered *L. camara* leaves was cold-macerated in ethanol (70%) and water (30%) for 72 h with periodic stirring. On the third day, leaf material was removed from solvents by filtration, and the filtrate was concentrated using a rotary evaporator (Buchi Labotechnik Rotavap R-210, Switzerland). The water was also removed by boiling on water bath at 60 °C (GFL 1032, Germany). The moist gummy solid extract with a final yield of 8.29% (w/w) was stored in an air-tight container and kept in a desiccator. The concentrated extract was referred to as *Lantana camara* extract (LCE) in this study.

### Experimental procedure

In this work, all drugs were freshly prepared before use. The design of the dosing regimen for the drugs donepezil and LCE were guided by previous work done by Zhang et al. (2013) and Millycent et al. (2017), respectively. The behavior of the animals was recorded and scored visually by a person blinded to the experimental groups using JWatcher Version 0.9 Built 2000-11-09.

### Ameliorative effect of LCE in scopolamine-induced memory impairment in zebrafish

**Figure 1.**
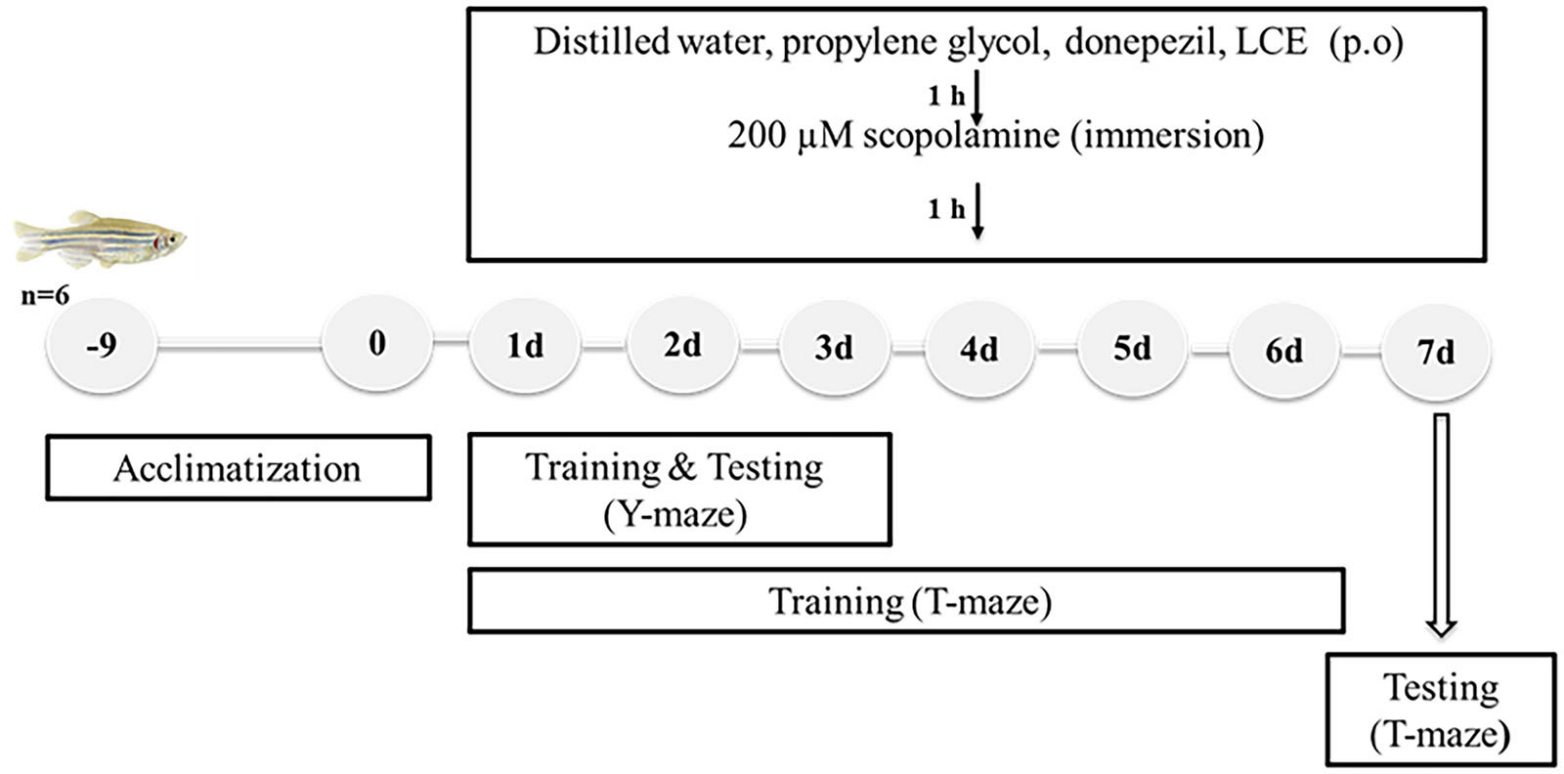
Schematic representation of scopolamine-induced memory impairment in zebrafish.

The ameliorative effect of LCE in scopolamine-induced memory-impaired zebrafish was determined using the T- and Y-maze tests to evaluate spatial short-term memory. Adult zebrafish were randomly selected into seven groups (*n* = 6, for each behavioral test) and given various treatments as shown in Table 1 below. First, fish, except the naïve, were anesthetized in tricaine (0.1 g/L) solution, after which various doses (5 µL maximum) of distilled water, propylene glycol, donepezil, or LCE were administered orally to the fish, using a 10 µL pipette. An hour after drug administration, each fish was directly placed in a 250 mL beaker filled with 100 mL of scopolamine solution (200 µM) for 1 h to induce memory impairment. Drug administration and testing of fish as performed in this work is shown in Figure 1.

**Table 1.** Summary of drug treatments in zebrafish.

Groups	Treatments
I	Untreated/normal animals (Naïve control)
II	Distilled water (5 µL, *p.o*) + 200 µM scopolamine immersion for 1 hr. (Scop. Dis. Control)
III	4% propylene glycol (5 µL, *p.o*) + 200 µM scopolamine immersion for 1 hr. (P.G Vehicle Control)
IV	0.65 mg/kg donepezil in 4% propylene glycol (5 µL, *p.o*) + 200 µM scopolamine immersion for 1 hr. (DPZ 0.65 mg/kg)
V-VII	10, 30 and 100 mg/kg LCE in 4% propylene glycol respectively (5 µL, *p.o*) + 200 µM scopolamine immersion for 1 hr. (LCE 10-100 mg/kg)

#### Zebrafish T-maze test

Spatial short-term memory of zebrafish was tested using an Offset cross maze configured as T-maze as previously described by Maddula et al. (2017) with slight modifications. Offset cross maze is an aquatic tank with a start box of 10 × 10 × 10 cm^3^ at the foot of the stem, a long arm of 70 × 10 × 10 cm^3^, and three short arms of 20 × 10 × 10 cm^3^ each. By closing off the top short arm, the offset cross maze was configured as a T-maze for the color-reward associative learning task. The two short arms (right and left arms) were covered with red and green colored sleeves respectively and the maze was filled with water up to 6 cm high. Each fish was given various treatments as shown in Table 1, daily, for 7 consecutive days. Fish were trained from days 1 to 6, immediately after the scopolamine immersion and finally tested on day 7, after the scopolamine immersion. Before the training session, zebrafish were initially habituated by placing them individually in the T-maze without colored sleeves for 1 min. Following habituation, the green and red colored sleeves were fitted around the short arms of the maze. During training, overnight fasted zebrafish were placed in the start box. After 1 min, the sliding door was opened and the fish was allowed to swim from it. After the exit of the fish, the door was closed. Next, the zebrafish was permitted to swim into the short arms and upon entering any of the short arms, a new sliding door at the junction of the two short arms and long arm was closed. The fish was now allowed to swim and observed for 4 min in the short arms. Fish were rewarded with food when they entered the green arm only. Zebrafish were returned to their respective tanks after training. On day 7, the same procedure as used in the training session was used for the test session, except that fish was not awarded food when it entered the green arm. The behavior of animals was recorded during the training and test sessions, after which the ‘latency to enter into the green arm’ and the ‘time spent in the green arm’ during the test session were scored. The percentage (%) of total time spent in the green arm was then calculated using the formula below:

(1) $$\%\;\mathrm{Total}\;\mathrm{time}\;=\frac{\mathrm{Time}\;\mathrm{spent}\;in\;\mathrm{green}\;\mathrm{arm}}{\mathrm{Total}\;\mathrm{time}\;\mathrm{allowed}\;in\;\mathrm{both}\;\mathrm{short}\;\mathrm{arms}\;(4\;\min)}\;\times \;100$$

#### Zebrafish Y-maze test

The Y-maze test previously described by Cognato et al. (2012) was also used to evaluate spatial short-term memory in zebrafish. The Y-maze is an aquatic tank consisting of three arms of equal sizes (25 cm long, 8 cm wide, and 15 cm high) and angled 120° apart from each other. Sliding doors were added to each arm to allow the closing of arms to create novel spaces. The arms of the Y-maze were randomly labeled as the start arm; in which fish started to explore (always opened), the novel arm; which was blocked during the training session and opened during the test session, and the other arm (always opened). Three different visual cues (squares, circles, and triangles) made of white paper cut were placed on the external walls of the maze, making them visible from inside the maze. To create a contrast between the fish and maze to aid video analysis of fish behavior, the external floor was covered with white paper. The remaining area of the external walls was covered with black paper and the maze was filled with water to a height of 8 cm. Zebrafish were given various treatments as shown in Table 1 for 3 consecutive days. Training in the maze was done each day immediately after the scopolamine immersion. During training, each fish was allowed to explore the start and other arms only for 5 min, with the novel arm closed. After the training, zebrafish were returned to their respective tanks. An hour after training, the test session started. To do this, the fish was placed back in the start arm and allowed to explore all three arms for 5 min. The behavior of animals was recorded during the training and test sessions after which the ‘time spent in the novel arm during the test session’ was scored. The percentage (%) of total time spent in the novel arm was calculated using the formula below:

(2) $$\%\;\mathrm{Total}\;\mathrm{time}=\frac{\mathrm{Time}\;\mathrm{spent}\;in\;\mathrm{novel}\;\mathrm{arm}}{\mathrm{Total}\;\mathrm{time}\;\mathrm{allowed}\;to\;\mathrm{explore}\;\mathrm{all}\;\mathrm{arms}\;(5\;\min)}\;\times \;100$$

### Ameliorative effect of LCE in scopolamine-induced memory impairment in mice

**Figure 2.**
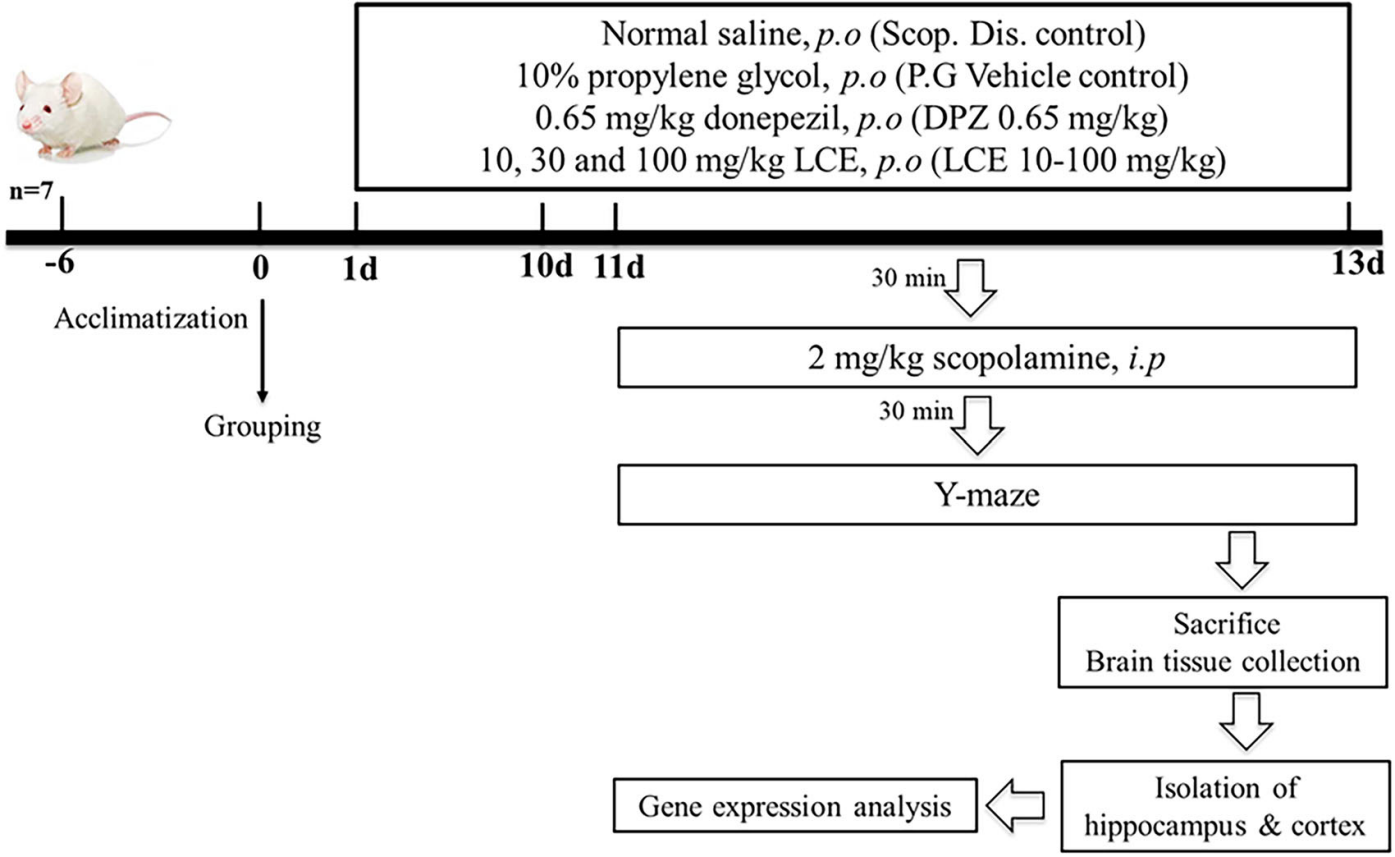
Schematic representation of scopolamine-induced memory impairment in mice.

The protective effect of LCE in scopolamine-induced memory impairment was assessed using ICR mice. Mice were put into seven groups (*n* = 7) and given various treatments for 13 days as presented in Table 2. Memory impairment was induced by 24 hourly intraperitoneal injections of 2 mg/kg scopolamine from days 11–13, 30 min after drug administration. Spatial memory of mice was assessed on days 11–13 using the Y-maze test, 30 min after the scopolamine administration. Drug treatment and testing of mice are shown in Figure 2 below.

**Table 2.** Summary of drug treatments in mice.

Groups	Treatments
I	Untreated/normal animals (Naïve control)
II	Normal saline (*p.o*) + i.p injection of 2 mg/kg scopolamine (Scop. Dis. control)
III	10 % propylene glycol (*p.o*) + i.p injection of 2 mg/kg scopolamine (P.G Vehicle control)
IV	0.65 mg/kg donepezil in 10% propylene glycol (*p.o*) + i.p injection of 2 mg/kg scopolamine (DPZ 0.65 mg/kg)
V-VII	10, 30 and 100 mg/kg LCE in 10% propylene glycol respectively (*p.o*) + i.p injection of 2 mg/kg scopolamine (LCE 10-100 mg/kg)

#### Y-maze spontaneous alternation test

The Y-maze spontaneous alternation test as described by Prieur and Jadavji (2019) was used to evaluate spatial short-term memory and general locomotor activity of mice. The maze is made of three equal arms (35 cm long, 13 cm high, and 7 cm wide), angled 120° apart from each other and labeled A, B, and C. Each mouse, naive to the maze was placed in the distal part of the arm labeled A, facing the center of the maze, and allowed to explore the maze undisturbed for 8 min whiles being recorded. The experimental arena was cleaned after testing each animal. An arm entry was made when a mouse move into an arm of the maze with all four paws. An alternation is a mouse entering all three arms of the maze consecutively. Alternations reflects spatial short-term memory (Kouémou et al. 2017) and the total number of arm entries reflects general locomotor activity (Parada-Turska and Turski 1990; Baral et al. 2015). The ‘number of alternations’ and the ‘total number of arm entries were scored from the recorded video. The percentage (%) spontaneous alternation was calculated as described by Karthivashan et al. (2018) using the formula below:

(3) $$\%\;\mathrm{Alternation}=\frac{\mathrm{Number}\;of\;\mathrm{Alternations}}{\left(\mathrm{Total}\;\mathrm{number}\;of\;\mathrm{Arm}\;\mathrm{Entries}-2\right)}\;\times \;100$$

### Brain tissue preparation

Immediately after the completion of the Y-maze test, mice were euthanized humanely through cervical dislocation, and whole brains were rapidly removed. The hippocampi and cortex were rapidly isolated on ice, snap-frozen in liquid nitrogen, and stored in a −80 °C refrigerator for RNA analysis.

### Gene expression analysis

The Guanidine thiocyanate extraction method was employed to extract total RNA from the mouse hippocampal and cortical regions using RNAsimple Total RNA Kit (TIANGEN, Beijing) according to the manufacturer’s instructions. Total RNA concentration and purity were measured using NanoDropTM Lite Spectrophotometer (Thermo Scientific, USA). Portions of total RNA (500 ng) were reverse transcribed to synthesize cDNA. Briefly, 6 μL RNA portions were added to 6 μL random primer master mix (2.4 μL random primer [30 μM], 3.6 μL of sterile RNAse-free ddH20) in PCR tubes. Next, the samples were vortexed to mix and incubated at 70 °C for 5 min in a thermocycler (SEEAMPTM PCR System, Seegene) for random primers to anneal to RNA. This was followed by the addition of 8 μL of a master mix (Reverse Transcriptase (0.5 μL M-MLV RT [H−] (200 U/μL), 4 μL M-MLV 5× buffer, 2 μL of 10 mM dNTPs, 1.5 μL sterile RNAse-free ddH20) to the PCR tubes. The contents were mixed thoroughly and incubated in the thermocycler to synthesize cDNA. Subsequently, the cDNA was employed in a quantitative real-time polymerase chain reaction (qRT-PCR) by adding 4 μL cDNA to 16 μL of qRT-PCR master mix (10 μL SYBR Green qPCR-Mix [TIANGEN, Beijing], 1 μL forward primer, 1 μL reverse primer, 4 μL RNase-free water) containing gene-specific primers (Table 3). The reaction was run in Rotor-Gene Q (Qiagen, Germantown, Maryland). Relative gene expression was calculated using the 2(-Delta Delta C (T)) method (Livak and Schmittgen 2001).

**Table 3.** Oligonucleotides.

Primers for qRT‐PCR	Sequence	Length	Tm (°C)
Ribosomal protein, large, P0 (Rplp0)	Forward 5'‐GGACCCGAGAAGACCTCCTT‐3' Reverse 5'‐GCACATCACTCAGAATTTCA‐3'	20 20	63 63
IL-1β	Forward 5'‐CCTTTTGACAGTGATGAGAATGAC‐3' Reverse 5'‐GAAGGTCCACGGGAAAGACAC‐3'	24 21	64 63
IL-6	Forward 5'‐AACCACGGCCTTCCCTACTT‐3' Reverse 5'‐TCCAGTTTGGTAGCATCCATCATTT‐3'	20 25	60 63
COX-2	Forward 5'‐TGAGCAACTATTCCAAACCAGC‐3' Reverse 5'‐GCACGTAGTCTTCGATCACTATC‐3'	22 23	60 63
TNF-α	Forward 5'‐GATCGGTCCCCAAAGAAGGGATG‐3' Reverse 5'‐TGATCTGAGTGTGAGGGTCTCG‐3'	23 22	63 64

### Gas chromatography-mass spectrometry analysis of LCE

Possible phytochemical components of hydroethanolic leaf extract of *Lantana camara* were investigated using gas chromatography-mass spectrometry (GC-MS). This analysis was carried out using a PerkinElmer GC Clarus 580 Gas Chromatograph interfaced to a Mass Spectrometer PerkinElmer (Clarus SQ 8 S) and also equipped with Elite-5MS (5% diphenyl/95% dimethyl poly siloxane) fused to a capillary column (30 × 0.25 μm ID × 0.25 μm DF). The oven temperature was serially set from 80 °C with progressive increments of 10 °C/min to 250 °C, then 5 °C/min to 280 °C and holding for 15 min at 280 °C. An electron ionization system was operated in electron impact mode with an ionization energy of 70 eV for GC-MS detection. High purity helium gas programmed at a constant flow rate of 1 mL/min, with an injection volume of 1 μL was used as the carrier gas. The temperature of the injector and the ion source was kept at 250 °C and 150 °C, respectively. Subsequently, the mass spectrum of the extract was generated at 70 eV with a scan interval of 0.1 s, and fragments from 45 to 450 Da were analyzed. The solvent delay and the total GC/MS running time were 0 to 2 min and 38 min, respectively. The mass-detector and the software employed to handle mass spectra and chromatograms used in this analysis were Turbo-Mass and Turbo-Mass ver-6.1.0, respectively. Interpretation of the mass-spectrum GC-MS was conducted using the database of the National Institute of Standards and Technology (NIST), which contains over 62,000 patterns.

### Statistical analysis

Data are presented as mean ± standard error of mean (SEM). Data were analyzed using one-way analysis of variance (ANOVA) followed by Tukey’s tests. All statistical analyses were performed using GraphPad for Windows version 8 (GraphPad Prism Software Inc., San Diego, CA, USA). P value < 0.05 was considered statistically significant.

## Results

*LCE ameliorated scopolamine-induced memory impairment in the zebrafish T-maze test*

### Latency to enter green arm

**Figure 3.**
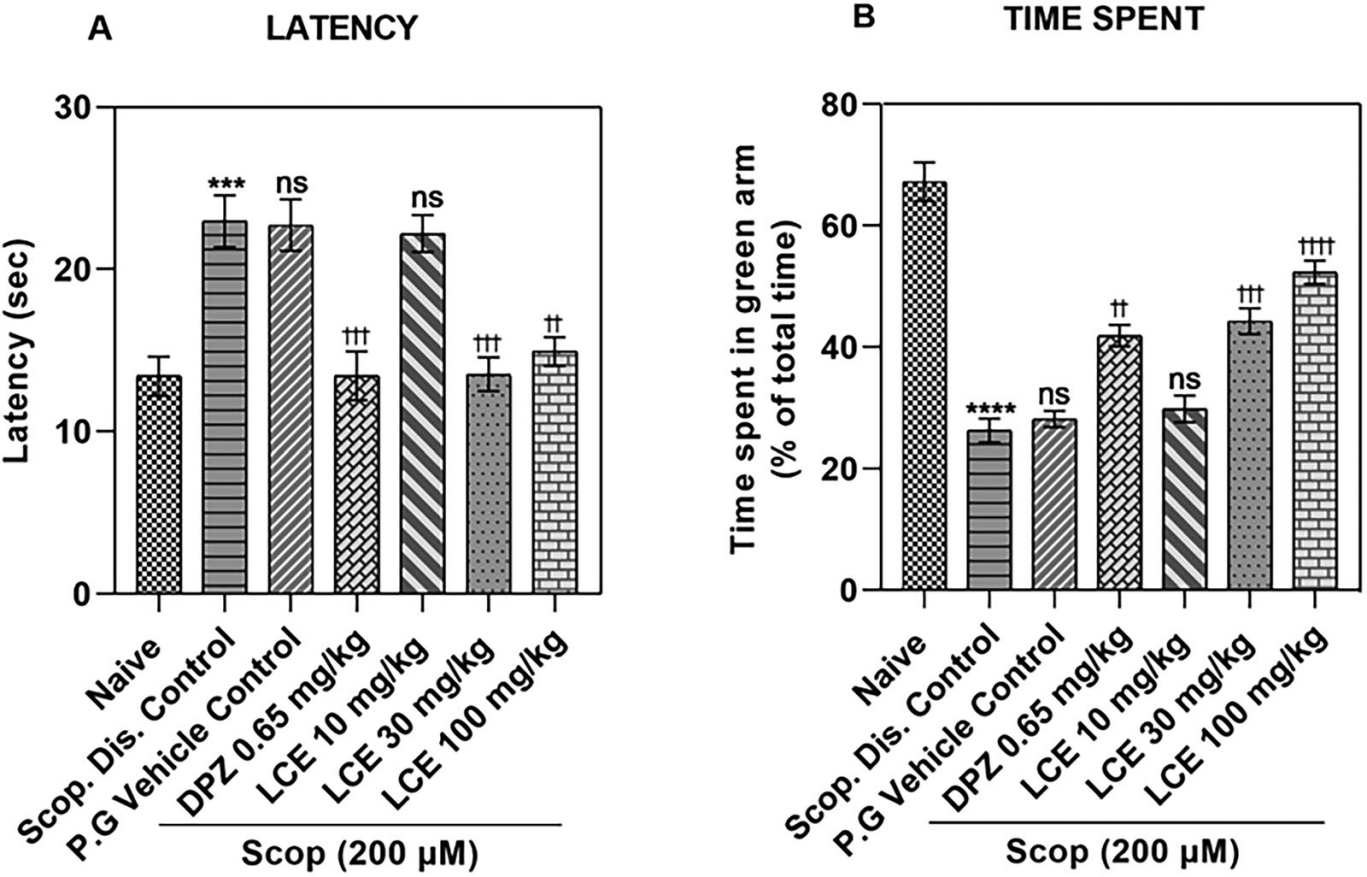
Effects of LCE on (A) Latency to enter the green arm, and (B) Time spent in the green arm in the zebrafish T-maze test. Results were presented as mean ± SEM (*n* = 6) using one-way ANOVA followed by Tukey’s multiple comparisons test. ***p < 0.001, ****p < 0.0001 compared to naïve control; ^††^*p* < 0.01, ^†††^*p* < 0.001, ^††††^*p* < 0.0001 compared to propylene glycol-treated group; ns - not significant.

Zebrafish with good memory have short latency times, while fish with defective memory show longer latency times. As shown in Figure 3(A), the disease control (Scop. Dis. Control) in this study showed significantly (*p* < 0.001) higher latency to entry (22.96 ± 1.60 s) when compared to the naïve group (13.40 ± 1.22 s). This was a 57.78 ± 3.64% increase in the latency, indicating scopolamine caused significant loss in memory. As expected, the scopolamine-induced increase in latency was not modulated by the vehicle propylene glycol (P.G Vehicle Control; 22.72 ± 1.59 s), meaning that propylene glycol does not affect the scopolamine response. Conversely, the standard drug group (DPZ) showed reduced latency to entry (13.43 ± 1.51 s), representing a 38.51 ± 4.23% decrease in latency when compared to the vehicle control, showing that this disease model responds to standard drug treatment. Although the lowest dose of LCE (10 mg/kg) did not affect the latency to entry (22.20 ± 1.14 s), the 30 and 100 mg/kg doses of LCE showed significantly lower latencies (13.52 ± 1.05 s and 14.93 ± 0.88 s respectively), representing a 38.48 ± 4.68% and 32.26 ± 6.71% decrease in latency to entry respectively when compared to the vehicle control group. This shows that prophylactic treatment with LCE provides some protection against scopolamine-induced memory loss in zebrafish T-maze test.

### Time spent in green arm

Zebrafish with deficient memory spend less time, while fish with good memory spend more time in the food containing (green) arm. The results in Figure 3(B) showed that the disease control (Scop. Dis. Control) spent less time in the green arm (26.24 ± 1.91%) compared to the naïve group (67.24 ± 3.20%), which is a 60.22 ± 3.98% drop, confirming a significant loss of memory. As was observed earlier, the scopolamine-induced memory loss was not affected by the vehicle propylene glycol (P.G Vehicle Control; 28.11 ± 1.32%), as there was no significant difference in the time spent in the green arm when compared to the disease control. However, the standard drug group (DPZ) spent more time (41.85 ± 1.77%) in the green arm which was a 31.89 ± 5.53% increase in the time spent when compared to the vehicle control. This confirms that this model responds to standard drug treatment. Just as was observed earlier, the lowest dose of the extract (LCE) was not effective. However, LCE at 30 and 100 mg/kg (44.23 ± 2.13% and 52.30 ± 1.94%, respectively) showed a higher percentage time spent in the green arm, representing 35.27 ± 5.45% and 45.69 ± 3.80% increase, respectively, compared to the vehicle control group. This agrees with the earlier observations on latency, confirming that LCE displays a protective effect in the zebrafish T-maze test.

#### LCE ameliorated scopolamine-induced memory impairment in the zebrafish Y-maze test

**Figure 4.**
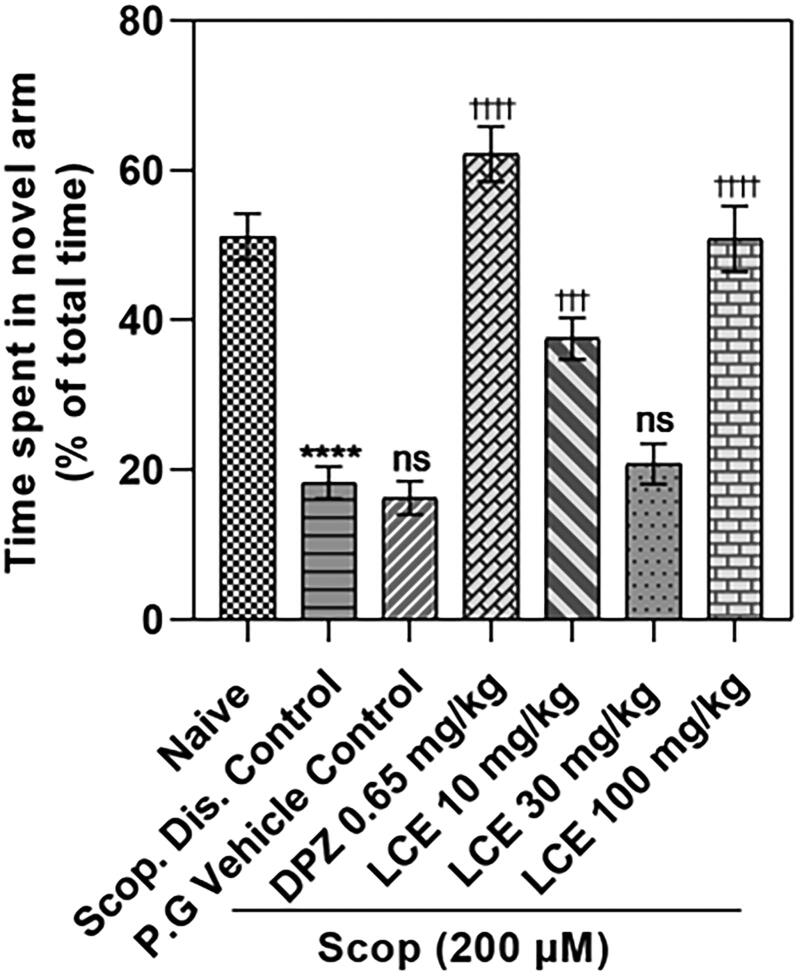
Effect of LCE on time spent in novel arm in the zebrafish Y-maze test. Results were presented as the mean ± SEM (*n* = 6) using one-way ANOVA followed by Tukey’s multiple comparisons test. ****p < 0.0001 compared to naïve control; ^†††^*p* < 0.001, ^††††^*p* < 0.0001 compared to propylene glycol-treated group; ns - not significant.

Zebrafish with good memory spend much more time in the novel arm compared to the start and other arms of the Y-maze. From the result (Figure 4), the disease control (Scop. Dis. Control) spent a lower proportion of time in the novel arm (18.32 ± 2.14%) compared to the naïve group (51.19 ± 3.06%). This is a 64.10 ± 4.16% decrease in time spent, confirming that scopolamine induces significant defects in the short-term memory of zebrafish. The scopolamine-induced memory loss was not affected by the vehicle propylene glycol (P.G Vehicle Control; 16.27 ± 2.22%), as there was no significant difference in the percentage of total time spent in the novel arm compared to the disease control (18.32 ± 2.14%). Donepezil group showed a remarkably higher (*p* < 0.0001) percentage of total time in the novel arm (62.17 ± 3.65%) compared to the 16.27 ± 2.22% time spent by the vehicle control group, which is a 74.00 ± 3.01% increase in percentage time spent. This further confirms that this zebrafish model responds to standard AD drug treatment. Differing from what was observed in the T-maze where LCE was effective at 30 and 100 mg/kg, LCE was effective at 10 and 100 mg/kg in this maze, with the animals spending 37.54 ± 2.78% and 50.84 ± 4.37% of their time respectively in the novel arm when compared to the vehicle control group (16.27 ± 2.22%). This corresponds to a 55.89 ± 5.70% and 68.21 ± 2.75% increase, respectively, in time spent. Thus, LCE did not show a clear dose-dependent pattern. Nevertheless, this observation supports the initial finding from the T-maze, confirming that LCE is protective against scopolamine-induced memory impairment in zebrafish.

## LCE ameliorated scopolamine-induced memory impairment in mice Y-maze test

### Percentage spontaneous alternation

Spontaneous alternation is a behavioral test for measuring exploratory behavior based on the willingness of rodents to explore a new environment. Normal rodents will prefer to experience a different arm of the maze than the one they visited on their previous entry, thus exhibiting higher spontaneous alternation in comparison to memory-deficient animals.

**Figure 5.**
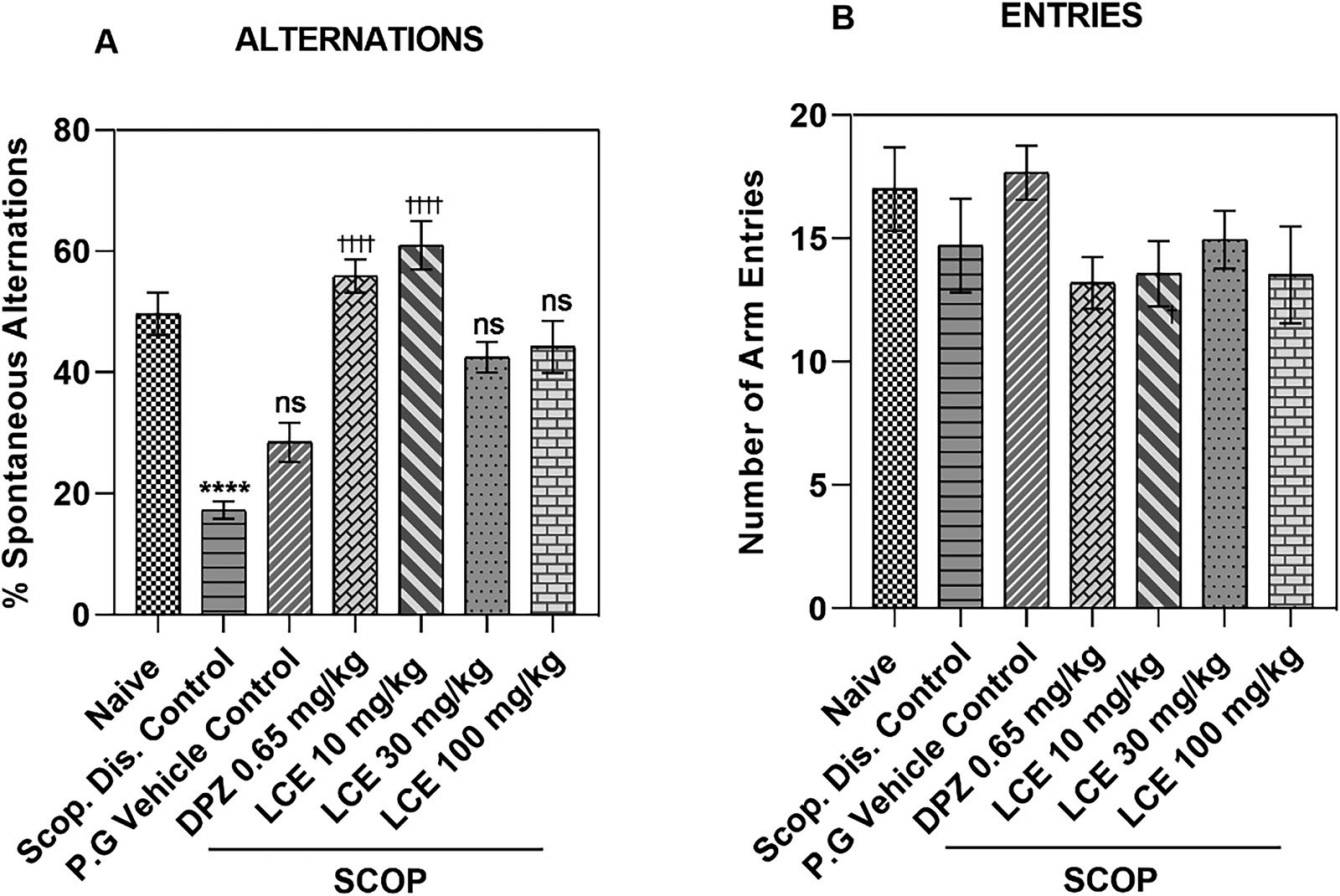
Effects of LCE on (A) Percentage spontaneous alternation, and (B) Number of arm entries in mice Y-maze test. Results were presented as the mean ± SEM (*n* = 7) using one-way ANOVA followed by Tukey’s multiple comparisons test. ****p < 0.0001 compared to naïve control; ^††††^ *p* < 0.0001 compared to propylene glycol-treated group; ns - not significant.

From the results (Figure 5(A)), the disease control (Scop. Dis. Control) showed a lower (*p* < 0.0001) percentage spontaneous alternation (17.26 ± 1.43%), compared to the naïve group (49.66 ± 3.49%), which is a 64.21 ± 4.15% decrease in percentage alternation. As anticipated, the propylene glycol vehicle (28.47 ± 3.25%) did not have any significant effect on the decrease in percentage alternations induced by scopolamine when compared to disease control. The donepezil-treated group (DPZ) showed a higher percentage spontaneous alternation (55.91 ± 2.72%) when compared to the vehicle control, representing a 47.17 ± 7.65% increase, showing that this model responds to standard drug treatment. LCE was only effective at the lowest dose (10 mg/kg), as it enhanced the spontaneous alternations to 60.98 ± 4.01%, which is a 52.89 ± 4.98% increase in spontaneous alternation when compared to the vehicle control. Albeit, these results also suggest that LCE offers some protection against scopolamine-induced memory impairment in mice.

### Total number of arm entries

The effect of drug treatment and experimental conditions on locomotion can influence general exploratory activity to confound the results in the behavioral analysis including the Y-maze. The total number of arm entries, a measure of locomotion (Parada-Turska and Turski 1990; Prieur and Jadavji 2019) was assessed in the Y-maze to determine whether treatment and experimental conditions affected the locomotion of animals. For the total number of arm entries (Figure 5(B)), no significant difference was observed in the disease control (Scop. Dis. Control; 14.71 ± 1.90) when compared to the naïve group (17.00 ± 1.69). The response in the vehicle control group (17.67 ± 1.09) was not significantly (*p* > 0.05) different from the disease control. Similarly, that of DPZ (0.65 mg/kg; 13.19 ± 1.05) and LCE (10, 30, 100 mg/kg; 13.57 ± 1.32, 14.95 ± 1.18, and 13.52 ± 1.97, respectively) were all not significantly (p > 0.05) different compared to the vehicle control group. This suggests that locomotor activity was neither affected by treatment nor experimental conditions. Hence the LCE effect on spontaneous alternation is likely due to the modulation of memory processing.

#### LCE ameliorated scopolamine-induced neuroinflammation in mice

Analysis of proinflammatory gene expression is one method of evaluating drug effect in neuroinflammation in AD (Abid et al. 2019). As AD pathology is initiated and is most severe in the hippocampus and cortex (Serrano-Pozo et al. 2011), the drug effect on proinflammatory gene expression was evaluated in mouse hippocampal and cortical tissues.

#### LCE altered proinflammatory gene expression in mice hippocampi

**Figure 6.**
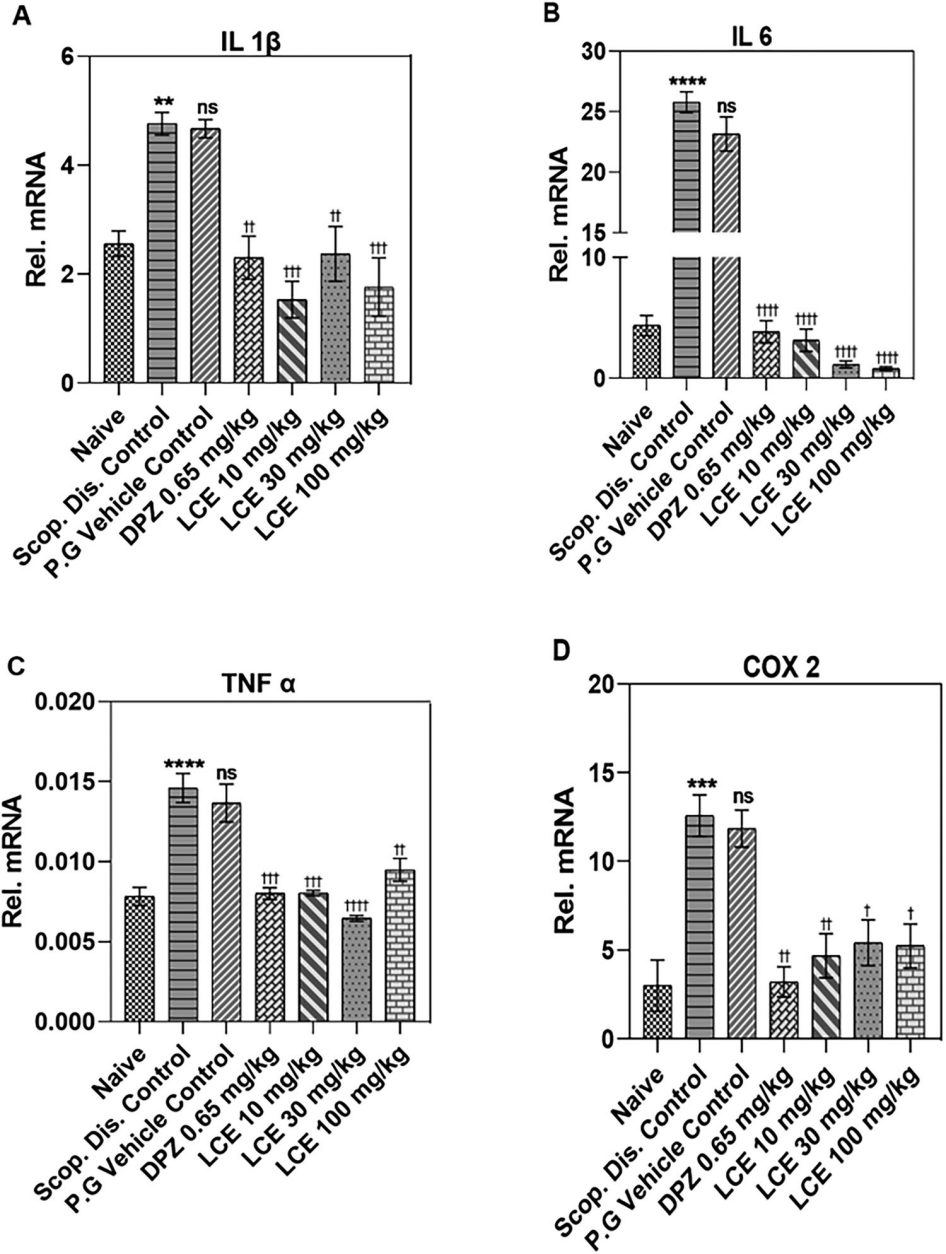
Effect of LCE in scopolamine-induced expression of IL-1β, IL-6, TNF-α and COX-2 in mouse hippocampi. Results are presented as mean ± standard error of mean (*n* = 7). Data analysis was performed using one-way ANOVA followed by Tukey’s multiple comparisons test. **p < 0.01, ***p < 0.001, ****p < 0.0001 compared to naïve control group; ^†^*p* < 0.05, ^††^*p* < 0.01, ^†††^*p* < 0.001, ^††††^*p* < 0.0001 compared to propylene glycol-treated group; ns- not significant.

Scopolamine significantly elevated the mRNA levels of proinflammatory cytokines (IL-1β, IL-6, TNF-α), and the proinflammatory enzyme COX-2 to 4.76 ± 0.19, 25.78 ± 1.18, 0.015 ± 0.001, and 12.58 ± 0.63, respectively, in the hippocampus (Figure 6(A–D)) when compared to the naïve control (2.56 ± 0.15, 4.36 ± 0.40, 0.008 ± 0.001, and 3.00 ± 0.77, respectively). IL-1β (89.46 ± 12.42%; Figure 6(A)) and IL-6 (519.10 ± 56.74%; Figure 6(B)) showed the lowest and highest percentage increase, respectively. This indicates that scopolamine-induced neuroinflammation in mice hippocampi. As expected, the vehicle did not alter the scopolamine-induced expression of these genes. However, donepezil significantly inhibited the scopolamine-induced increase in mRNA expression for all the selected genes, with the highest inhibitory effect on IL-6 (83.27 ± 2.49%; Figure 6(B)). Likewise, LCE inhibited scopolamine-induced elevated mRNA expression for all the selected genes with its most significant inhibition on IL-6 expression (89.70 ± 1.52%, 95.12 ± 0.58%, and 96.53 ± 0.31%; 10, 30, and 100 mg/kg, respectively) when compared to vehicle control (Figure 6(B)). This suggests that both donepezil and LCE attenuated scopolamine-induced neuroinflammation in mice hippocampi.

#### LCE altered proinflammatory gene expression in mouse cerebral cortex

**Figure 7.**
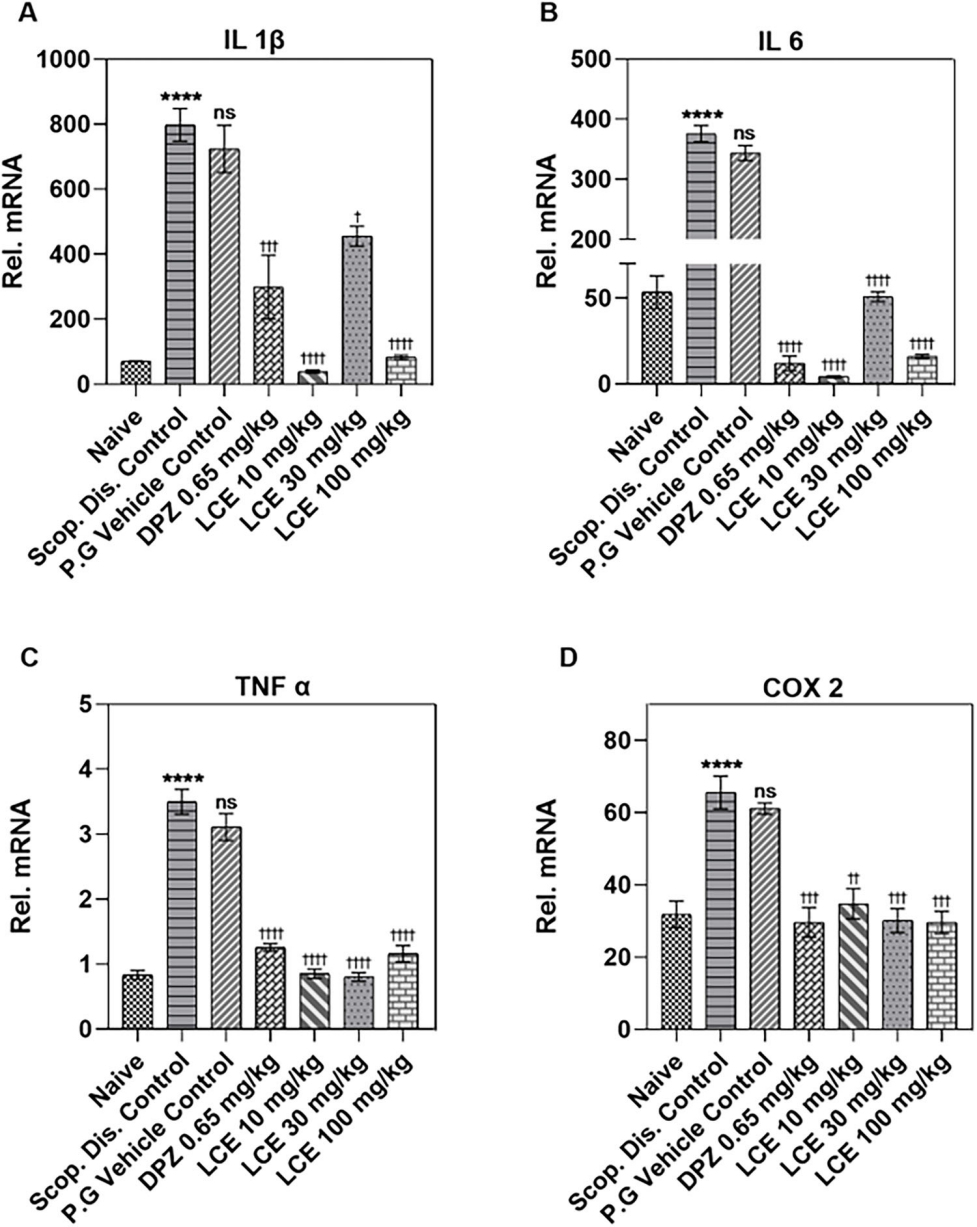
Effect of LCE in scopolamine-induced expression of IL-1β, IL-6, TNF-α, and COX-2 in mouse cerebral cortex. Results are presented as mean ± standard error of mean (*n* = 7). Data analysis was performed using one-way ANOVA followed by Tukey’s multiple comparisons test. ****p < 0.0001 compared to naïve control group; ^†^*p* < 0.05, ^††^*p* < 0.01, ^†††^*p* < 0.001, ^††††^*p* < 0.0001 compared to propylene glycol-treated group; ns- not significant.

The pattern of the inflammatory response in the cerebral cortex (Figure 7) is similar to what was observed for the hippocampus (Figure 6). Here also, scopolamine significantly (*p* < 0.0001) elevated mRNA levels of the proinflammatory cytokines (IL-1β, IL-6, TNF-α), and the proinflammatory enzyme COX-2 to 797.50 ± 31.35, 375.70 ± 12.96, 3.50 ± 0.13, and 65.57 ± 3.80, respectively. Predictably, the vehicle propylene glycol did not affect the scopolamine-induced inflammatory gene expression when compared to the disease controls. On the other hand, donepezil significantly repressed the scopolamine-induced gene (IL-1β, IL-6, TNF-α, and COX-2) expression, with the highest inhibitory effect on IL-6 (96.45 ± 0.76%; Figure 7(B)). Similarly, LCE at all the doses tested remarkably reduced the mRNA expression of IL-1β, IL-6, TNF-α, and COX-2 with maximum inhibition on IL-6 (98.74 ± 0.11%; 10 mg/kg; Figure 7(B)), showing that LCE inhibits neuroinflammation in the cerebral cortex. The current analysis shows that although the extract caused a significant inhibition of cytokine gene expression at all the doses tested, this did not correlate with the behavioral response.

#### GC-MS analysis of LCE

**Figure 8.**
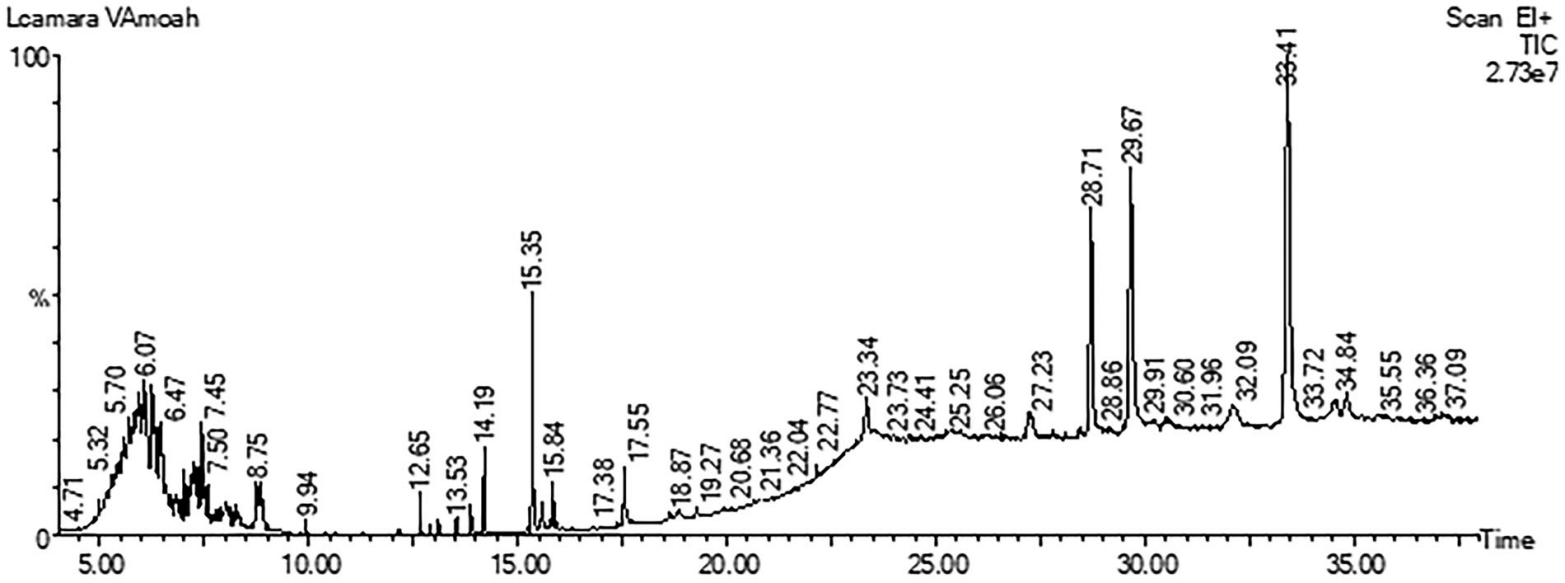
Gas chromatogram of hydroethanolic leaf extract of *Lantana camara.*

**Figure 9.**
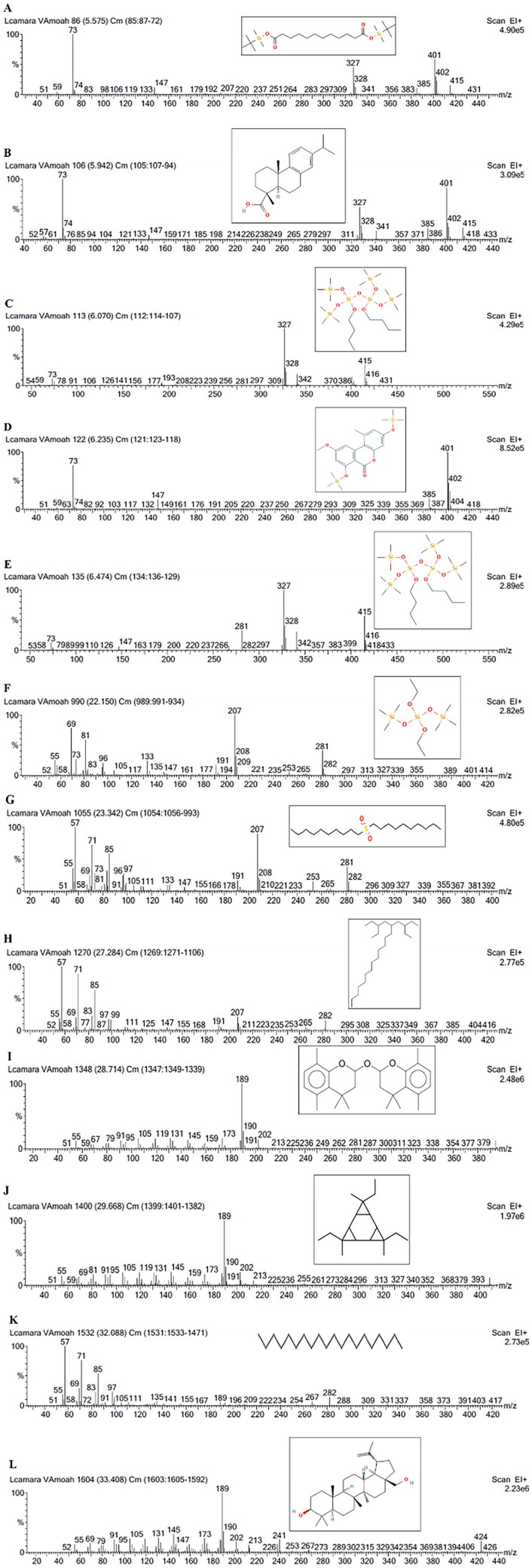
Mass spectra of hydroethanolic leaf extract of *Lantana camara.*

The resolution of LCE with GC-MS resulted in the identification of 12 chemical constituents (Figure 8, Table 4). Principally, GC-MS analysis revealed the presence of dodecanedioic acid, *bis*(*tert*-butyldimethylsilyl) ester, dehydroabietic acid, *bis*[(trimethylsilyl)oxy]heptanetetrasiloxane, 3,7-*bis*[(trimethylsilyl)oxy]-9-methoxy-1-methyl(6H)dibenzo[*b*,*d*]pyran-6-one, eicosane, botulin, di-*n*-decylsulfone, *bis*(trimethylsilyl) diethyl silicate, among others (Figure 9).

**Table 4.** List of compounds present in hydroethanolic leaf extract of *Lantana camara* identified by GC-MS analysis.

No.	RT	%Area	Compound
1	5.575	2.620	Dodecanedioic acid, *bis*(*tert*-butyldimethylsilyl) ester
2	5.942	3.741	Dehydroabietic acid
3	6.070	3.267	*Bis*[(trimethylsilyl)oxy]heptanetetrasiloxane
4	6.235	2.709	3,7-*Bis*[(trimethylsilyl)oxy]-9-methoxy-1-methyl(6H)dibenzo[b,d]pyran-6-one
5	6.474	2.128	3,5-Dibutoxy-1,1,1,7,7,7-hexamethyl-3,5-bis(trimethylsiloxy)tetrasiloxane
6	22.150	2.424	Silicic acid, diethyl *bis*(trimethylsilyl) ester
7	23.342	14.852	Di-*n*-decylsulfone
8	27.284	19.467	Octadecane, 3-ethyl-5-(2-ethylbutyl)-
9	28.714	4.988	Chromane,4,4,5,8-tetramethyl-2-[(4,4,5,8-tetramethyl-3,4-dihydro-2H-2-chromenyl)oxy]
10	29.668	7.834	Tetracyclo[6.1.0.0(2,4).0(5,7)]nonane, 3,6,9-triethyl-3,6,9-trimethyl-
11	32.088	3.220	Eicosane
12	33.408	7.586	Betulin

GC-MS: gas chromatography mass spectrometry.

## Discussion

Results from the behavioral and gene expression analysis performed in this investigation showed that prophylactic treatment with LCE protects against scopolamine-induced memory impairment in both zebrafish and mouse models of AD.

AD affects short-term memory at the early stages (Götz et al. 2018) which progresses ultimately to loss of long-term memory (Jahn 2013). In addition to memory impairments, other cognitive domains such as complex attention, executive functioning, language, perceptual-motor function, and social cognition are also impaired (Commins and Kirby 2019). Experimentally, drug response in AD can be studied in various animal models that mimic alterations in behavioral and cellular processes. Short-term memory (Suganthy et al. 2016) and neuroinflammation (Li et al. 2011) are examples of behavioral and cellular processes, respectively, that can be evaluated to determine drug effects in AD. Drug effects on behavioral alterations can be assessed in popular tests such as the Y- and T-mazes that measure spatial memory deficit (Suganthy et al. 2016; Ardestani et al. 2017) to test short-term memory (Suganthy et al. 2016). These tests have been used to characterize learning and memory in zebrafish (Cognato et al. 2012; Maddula et al. 2017) and rodents (Ardestani et al. 2017; Prieur and Jadavji 2019) in both transgenic (Ardestani et al. 2017) and a non-genetic model like scopolamine (Yadang et al. 2020; Callahan et al. 2021), making our selected battery of tests scientifically sound for this investigation.

Scopolamine is a cholinergic receptor antagonist that interferes with cholinergic transmission to induce performance impairment of learning and memory that mainly affects short-term memory (Sambeth et al. 2007; Klinkenberg and Blokland 2010). It provides a simple and quick way for testing the cognition-enhancing properties of new drugs, supported by the popular cholinergic hypothesis, which relates age-related deterioration in cognitive functions to a decrease in the integrity of cholinergic neurotransmission, as seen in geriatric memory dysfunction (Klinkenberg and Blokland 2010). Indeed, the well-established treatments for AD, rivastigmine, galantamine, and donepezil modulate cholinergic transmission (Anand and Singh 2013; Andrieu et al. 2015; Godyń et al. 2016). One major drawback of scopolamine use is the possibility of scopolamine affecting the locomotor activity of animals (Klinkenberg and Blokland 2010). This warranted the assessment of locomotor activity using the number of arm entries (Pellow et al. 1985; Parada-Turska and Turski 1990) as was carried out in this work using the Y-maze test in mice. As the number of arm entries did not change significantly, it can be safely concluded that the drug treatment and the experimental environment did not affect locomotor activity.

Scopolamine also induces neuroinflammation in the brain (Cheon et al. 2021), an event reported to facilitate and exacerbate both Aβ plaques and NFTs formation, the two core pathologies associated with human AD development (Kinney et al. 2018). Brain inflammation is described to have a dual role, as neuroprotective during an acute-phase response, but becomes detrimental when prolonged (Kinney et al. 2018), with a characteristic increase in proinflammatory mediators including proinflammatory cytokines such as IL-1β (Blum-Degen et al. 1995; Akiyama et al. 2000), IL-6 (Blum-Degen et al. 1995) and TNF-α (Fillit et al. 1991; Akiyama et al. 2000) and enzymes like COX-2 (Hoozemans et al. 2001, 2002), which in turn triggers several potentially harmful effects that promote AD pathologies.

TNF-α is prominently implicated in AD development (Perry et al. 2001). Increased expression of brain TNF-α activates neuronal apoptosis (Varfolomeev and Ashkenazi 2004; Li et al. 2004) *via* several mechanisms that lead to neurodegeneration including severe degeneration of cholinergic neurons in cortical and hippocampal areas of the brain (Whitehouse et al. 1982; Coyle et al. 1983), which is one of the most fundamental and consistent features that contribute to AD in humans (Whitehouse et al. 1981). Not surprisingly, TNF-α blocker biologics have shown some promise in AD management (Torres-Acosta et al. 2020).

IL-1β is chronically upregulated in human AD (Shaftel et al. 2008) but knowledge about the molecular mechanisms by which IL-1β promotes AD is still nascent. However, *in vivo* (Brugg et al. 1995) and *in vitro* (Liao et al. 2004) studies have shown that IL-1β increases the expression of Aβ plaques, a toxic oligomer that causes neurodegeneration by apoptosis mediated by caspases (Iadanza et al. 2018) and contribute among other events to cholinergic dysfunction (Li et al. 2000) leading to memory loss. IL-6 levels rise in early AD pathologies and hence proposed as a serum biomarker for AD (Bermejo et al. 2008). Amongst other events, it enhances Aβ (Qiu and Gruol 2003) and tau protein (Quintanilla et al. 2004) induced neuronal damage to contribute to memory impairment. Neuronal COX-2 expression is increased in the early stages of AD (Hoozemans et al. 2001, 2002; Yermakova and O'Banion, 2001; Ho et al. 2001) suggesting they have a role in early AD pathologies. It promotes neuroinflammation by stimulating IL-1β, TNF-α and IL-6 expression (Wang et al.^2021^) and Aβ plaques accumulation in neurons (Nilsson et al. 2013) to cause neuronal damage and memory loss. Thus, the potent inhibition of these proinflammatory genes in the brain of mice shows that LCE also modulated cellular processes involved in AD pathology, confirming the improvement in the behavioral tests.

In this work, our GC-MS analysis identified several compounds such as dehydroabietic acid, silicic acid, dodecanedioic acid, betulin, and diethyl *bis*(trimethylsilyl) ester which have also been found to possess anti-inflammatory, antioxidant (Kukuia et al. 2022; Liu et al. 2020), and neuroprotective properties (Sierra et al. 2022) through the inhibition of IL-1β, IL-6, and TNF-α, leading to the suppression of microglia activation during neuroinflammation (Sierra et al. 2022).

As interesting as the current findings are, it is important to state that we did not observe a clear correlation between the dose of extract and the magnitude of the biological response. The extract was effective at high doses (30 and 100 mg/kg) in certain instances (zebrafish T and Y maze) or produced a U-shaped effect (10 and 100 mg/kg) in the mouse Y-maze test, although there was potent inhibition of proinflammatory cytokine gene expression in mouse brain tissue at all the doses (10, 30, and 100 mg/kg) tested. This phenomenon is common in natural product research involving whole animals (Guimarães et al. 1990; Nazario et al. 2015). Crude extracts from medicinal plants usually contain several compounds that can differentially interact with the complex biological systems of animals, resulting in different patterns of responses in different experimental environments. But despite the lack of a clear dose-response relationship, the observation that the extract has an ameliorative effect in both zebrafish (non-mammalian) and mouse (mammalian) models shows that the current finding is likely a general biological response in vertebrates that warrants further investigations.

The mechanism by which LCE produces its ameliorating effect was beyond this investigation. However, enhanced cholinergic transmission may have contributed significantly to the LCE response, as *L. camara* is previously reported to show acetylcholinesterase (AChE) inhibiting activities (Vinutha et al. 2007; Nour et al. 2014). This is the very mechanism by which established AD drugs in clinical use, rivastigmine, galantamine, and donepezil exhibit their therapeutic effects (Anand and Singh 2013; Andrieu et al. 2015; Godyń et al. 2016). Additionally, *L. camara* leaf was reported by different investigators to exhibit anti-inflammatory effects (Ashal et al. 2020) *via* suppression of the proinflammatory transcription factor NF-κB. This NF-κB inhibiting effect was attributed to the presence of the compounds 14 (3b, 22b-di (2-(2-(2, 6-dichlorophenylamino) phenyl) acetoyloxy)-olean-12-en-28-oic acid) and dehydroabietic acid present in the leaf (Kang et al. 2008; Suthar et al. 2014). The leaf also contained dodecanedioic acid, bis(tert-butyldimethylsilyl) ester which is present in some medicinal plants associated with an increase in prefrontal cortex dendritic spine density (Kukuia et al. 2022). However, further investigations are needed to understand the exact mechanism by which LCE inhibited the scopolamine effect in these experimental animals.

The potent inhibition of pro-inflammatory genes in the brain shows that LCE also modulated cellular processes involved in AD pathology, further confirming the improvement in the behavioral tests. As LCE provided significant protection in non-mammalian (zebrafish) and mammalian (mice) vertebrate models of AD, it may hold some potential for AD drug development, and hence qualifies as a candidate plant for further investigations, including investigations in human subjects. One limitation of this work is the need for protein analysis to further confirm the impact of modulation of inflammatory gene expression by LCE on its ameliorative effect.

## Data Availability

The data that support the findings of this study are available from the corresponding author, [GA], upon reasonable request.

## References

[CIT0001] Abid NB, Naseer MI, Kim MO. 2019. Comparative gene-expression analysis of Alzheimer’s Disease progression with aging in transgenic mouse model. IJMS. 20(5):1219. 30862043 10.3390/ijms20051219 PMC6429175

[CIT0002] Adeniyi A, Asase A, Ekpe PK, Asitoakor BK, Adu-Gyamfi A, Avekor PY. 2018. Ethnobotanical study of medicinal plants from Ghana; confirmation of ethnobotanical uses, and review of biological and toxicological studies on medicinal plants used in Apra Hills Sacred Grove. J Herb Med. 14:76–87.

[CIT0003] Akiyama H, Barger S, Barnum S, Bradt B, Bauer J, Cole GM, Cooper NR, Eikelenboom P, Emmerling M, Fiebich BL, et al. 2000. Inflammation and Alzheimer’s disease. Neurobiol Aging. 21(3):383–421. 10858586 10.1016/s0197-4580(00)00124-x PMC3887148

[CIT0004] Alasmari F, Alshammari MA, Alasmari AF, Alanazi WA, Alhazzani K. 2018. Neuroinflammatory cytokines induce amyloid beta neurotoxicity through modulating amyloid precursor protein levels/metabolism. Biomed Res Int. 2018:3087475. 30498753 10.1155/2018/3087475 PMC6222241

[CIT0005] Alonso A, Del C, Li B, Grundke-Iqbal I, Iqbal K. 2008. Mechanism of tau-induced neurodegeneration in Alzheimer disease and related tauopathies. Curr Alzheimer Res. 5(4):375–384. 18690834 10.2174/156720508785132307

[CIT0006] Alzheimer’s Association. 2021. 2021 Alzheimer’s disease facts and figures. Alzheimers Dement. 17:327–406. 33756057 10.1002/alz.12328

[CIT0008] Anand P, Singh B. 2013. A review on cholinesterase inhibitors for Alzheimer’s disease. Arch Pharm Res. 36(4):375–399. 23435942 10.1007/s12272-013-0036-3

[CIT0009] Andrieu S, Coley N, Lovestone S, Aisen PS, Vellas B. 2015. Prevention of sporadic Alzheimer’s disease: lessons learned from clinical trials and future directions. Lancet Neurol. 14(9):926–944. 26213339 10.1016/S1474-4422(15)00153-2

[CIT0010] Ardestani PM, Evans AK, Yi B, Nguyen T, Coutellier L, Shamloo M. 2017. Modulation of neuroinflammation and pathology in the 5XFAD mouse model of Alzheimer’s disease using a biased and selective beta-1 adrenergic receptor partial agonist. Neuropharmacology. 116:371–386. 28089846 10.1016/j.neuropharm.2017.01.010 PMC5385159

[CIT0011] Ashal TF, Ifora I, Oktavia S. 2020. Potential anti-inflammatory effects of *Lantana camara* L. Int Res J Pharm Med Sci. 3(6):1–4.

[CIT0012] Baral S, Cho DH, Pariyar R, Yoon CS, Chang BY, Kim DS, Cho HK, Kim SY, Oh H, Kim YC, et al. 2015. The ameliorating effect of myrrh on scopolamine-induced memory impairments in mice. Evid Based Complement Alternat Med. 2015:925432. 26635888 10.1155/2015/925432 PMC4655272

[CIT0013] Bermejo P, Martín-Aragón S, Benedí J, Susín C, Felici E, Gil P, Ribera JM, Villar AM. 2008. Peripheral levels of glutathione and protein oxidation as markers in the development of Alzheimer’s disease from mild cognitive impairment. Free Radic Res. 42(2):162–170. 18297609 10.1080/10715760701861373

[CIT0014] Blasko I, Stampfer-Kountchev M, Robatscher P, Veerhuis R, Eikelenboom P, Grubeck-Loebenstein B. 2004. How chronic inflammation can affect the brain and support the development of Alzheimer’s disease in old age: the role of microglia and astrocytes. Aging Cell. 3(4):169–176. 15268750 10.1111/j.1474-9728.2004.00101.x

[CIT0015] Blum-Degen D, Müller T, Kuhn W, Gerlach M, Przuntek H, Riederer P. 1995. Interleukin-1 beta and interleukin-6 are elevated in the cerebrospinal fluid of Alzheimer’s and *de novo* Parkinson’s disease patients. Neurosci Lett. 202(1-2):17–20. 8787820 10.1016/0304-3940(95)12192-7

[CIT0017] Brugg B, Dubreuil YL, Huber G, Wollman EE, Delhaye-Bouchaud N, Mariani J. 1995. Inflammatory processes induce beta-amyloid precursor protein changes in mouse brain. Proc Natl Acad Sci U S A. 92(7):3032–3035. 7708769 10.1073/pnas.92.7.3032 PMC42353

[CIT0018] Callahan PM, Terry AV, Peitsch MC, Hoeng J, Koshibu K. 2021. Differential effects of alkaloids on memory in rodents. Sci Rep. 11(1):9843. 33972592 10.1038/s41598-021-89245-w PMC8110766

[CIT0019] Chen WN, Yeong KY. 2020. Scopolamine, a toxin-induced experimental model, used for research in Alzheimer’s disease. CNS Neurol Disord Drug Targets. 19(2):85–93. 32056532 10.2174/1871527319666200214104331

[CIT0020] Cheon SY, Koo B-N, Kim SY, Kam E, Nam J, Kim E. 2021. Scopolamine promotes neuroinflammation and delirium-like neuropsychiatric disorder in mice. Sci Rep. 11(1):8376. 33863952 10.1038/s41598-021-87790-y PMC8052461

[CIT0021] Cognato GdP, Bortolotto JW, Blazina AR, Christoff RR, Lara DR, Vianna MR, Bonan CD. 2012. Y-Maze memory task in zebrafish (*Danio rerio*): the role of glutamatergic and cholinergic systems on the acquisition and consolidation periods. Neurobiol Learn Mem. 98(4):321–328. 23044456 10.1016/j.nlm.2012.09.008

[CIT0022] Coyle JT, Price DL, DeLong MR. 1983. Alzheimer’s disease: a disorder of cortical cholinergic innervation. Science. 219(4589):1184–1190. 6338589 10.1126/science.6338589

[CIT0023] Commins S, Kirby BP. 2019. The complexities of behavioral assessment in neurodegenerative disorders: a focus on Alzheimer’s disease. Pharmacol Res. 147:104363. 31374247 10.1016/j.phrs.2019.104363

[CIT0024] Directive 2010/63/EU of the European Parliament and of the Council of 22 September 2010 on the protection of animals used for scientific purposes (Text with EEA relevance). 2010. Official Journal of the European Union

[CIT0025] Dorey E, Chang N, Liu QY, Yang Z, Zhang W. 2014. Apolipoprotein E, amyloid-beta, and neuroinflammation in Alzheimer’s disease. Neurosci Bull. 30(2):317–330. 24652457 10.1007/s12264-013-1422-z PMC5562666

[CIT0026] Ferreira-Vieira TH, Guimaraes IM, Silva FR, Ribeiro FM. 2016. Alzheimer’s disease: targeting the cholinergic system. Curr Neuropharmacol. 14(1):101–115. 26813123 10.2174/1570159X13666150716165726 PMC4787279

[CIT0027] Fillit H, Ding WH, Buee L, Kalman J, Altstiel L, Lawlor B, Wolf-Klein G. 1991. Elevated circulating tumor necrosis factor levels in Alzheimer’s disease. Neurosci Lett. 129(2):318–320. 1745413 10.1016/0304-3940(91)90490-k

[CIT0028] Godyń J, Jończyk J, Panek D, Malawska B. 2016. Therapeutic strategies for Alzheimer’s disease in clinical trials. Pharmacol Rep. 68(1):127–138. 26721364 10.1016/j.pharep.2015.07.006

[CIT0029] Götz J, Bodea L-G, Goedert M. 2018. Rodent models for Alzheimer disease. Nat Rev Neurosci. 19(10):583–598. 30194347 10.1038/s41583-018-0054-8

[CIT0030] Guimarães FS, Chiaretti TM, Graeff FG, Zuardi AW. 1990. Antianxiety effect of cannabidiol in the elevated plus-maze. Psychopharmacology. 100(4):558–559. 1969666 10.1007/BF02244012

[CIT0031] Ho L, Purohit D, Haroutunian V, Luterman JD, Willis F, Naslund J, Buxbaum JD, Mohs RC, Aisen PS, Pasinetti GM. 2001. Neuronal cyclooxygenase 2 expression in the hippocampal formation as a function of the clinical progression of Alzheimer disease. Arch Neurol. 58(3):487–492. 11255454 10.1001/archneur.58.3.487

[CIT0032] Hoozemans JJ, Brückner MK, Rozemuller AJ, Veerhuis R, Eikelenboom P, Arendt T. 2002. Cyclin D1 and cyclin E are co-localized with cyclo-oxygenase 2 (COX-2) in pyramidal neurons in Alzheimer disease temporal cortex. J Neuropathol Exp Neurol. 61(8):678–688. 12152783 10.1093/jnen/61.8.678

[CIT0033] Huang Y, Mucke L. 2012. Alzheimer mechanisms and therapeutic strategies. Cell. 148(6):1204–1222. 22424230 10.1016/j.cell.2012.02.040 PMC3319071

[CIT0034] Hoozemans JJ, Rozemuller AJ, Janssen I, De Groot CJ, Veerhuis R, Eikelenboom P. 2001. Cyclooxygenase expression in microglia and neurons in Alzheimer’s disease and control brain. Acta Neuropathol. 101(1):2–8. 11194936 10.1007/s004010000251

[CIT0035] Iadanza MG, Jackson MP, Hewitt EW, Ranson NA, Radford SE. 2018. A new era for understanding amyloid structures and disease. Nat Rev Mol Cell Biol. 19(12):755–773. 30237470 10.1038/s41580-018-0060-8 PMC7617691

[CIT0036] Jahn H. 2013. Memory loss in Alzheimer’s disease. Dialogues Clin Neurosci. 15(4):445–454. 24459411 10.31887/DCNS.2013.15.4/hjahn PMC3898682

[CIT0037] Kang MS, Hirai S, Goto T, Kuroyanagi K, Lee JY, Uemura T, Ezaki Y, Takahashi N, Kawada T. 2008. Dehydroabietic acid, a phytochemical, acts as ligand for PPARs in macrophages and adipocytes to regulate inflammation. Biochem Biophys Res Commun. 369(2):333–338. 18267111 10.1016/j.bbrc.2008.02.002

[CIT0038] Kar S, Issa AM, Seto D, Auld DS, Collier B, Quirion R. 1998. Amyloid beta-peptide inhibits high-affinity choline uptake and acetylcholine release in rat hippocampal slices. J Neurochem. 70(5):2179–2187. 9572306 10.1046/j.1471-4159.1998.70052179.x

[CIT0039] Karthivashan G, Park SY, Kweon MH, Kim J, Haque ME, Cho DY, Kim IS, Cho EA, Ganesan P, Choi DK. 2018. Ameliorative potential of desalted *Salicornia europaea* L. extract in multifaceted Alzheimer’s-like scopolamine-induced amnesic mice model. Sci Rep. 8(1):7174. 29740000 10.1038/s41598-018-25381-0 PMC5940894

[CIT0040] Kinney JW, Bemiller SM, Murtishaw AS, Leisgang AM, Salazar AM, Lamb BT. 2018. Inflammation as a central mechanism in Alzheimer’s disease. Alzheimers Dement. 4:575–590. 10.1016/j.trci.2018.06.014 PMC6214864 30406177

[CIT0041] Klinkenberg I, Blokland A. 2010. The validity of scopolamine as a pharmacological model for cognitive impairment: a review of animal behavioral studies. Neurosci Biobehav Rev. 34(8):1307–1350. 20398692 10.1016/j.neubiorev.2010.04.001

[CIT0042] Kouémou NE, Taiwe GS, Moto FCO, Pale S, Ngoupaye GT, Njapdounke JSK, Nkantchoua GCN, Pahaye DB, Bum EN. 2017. Nootropic and neuroprotective effects of *Dichrocephala integrifolia* on scopolamine mouse model of Alzheimer’s disease. Front Pharmacol. 8:847. 29209218 10.3389/fphar.2017.00847 PMC5702348

[CIT0043] Kukuia KKE, Appiah F, Dugbartey GJ, Takyi YF, Amoateng P, Amponsah SK, Adi-Dako O, Koomson AE, Ayertey F, Adutwum-Ofosu KK. 2022. Extract of *Mallotus oppositifolius* (Geiseler) Müll. Arg. increased prefrontal cortex dendritic spine density and serotonin and attenuated parachlorophenylalanine-aggravated aggressive and depressive behaviors in mice. Front Pharmacol. 13:962549. 36386158 10.3389/fphar.2022.962549 PMC9649488

[CIT0045] Lee DC, Rizer J, Hunt JB, Selenica ML, Gordon MN, Morgan D. 2013. Review: experimental manipulations of microglia in mouse models of Alzheimer’s pathology: activation reduces amyloid but hastens tau pathology. Neuropathol Appl Neurobiol. 39(1):69–85. 23171029 10.1111/nan.12002 PMC4300851

[CIT0047] Liao YF, Wang BJ, Cheng HT, Kuo LH, Wolfe MS. 2004. Tumor necrosis factor-α, interleukin-1β, and interferon-γ stimulate γ-secretase-mediated cleavage of amyloid precursor protein through a JNK-dependent MAPK pathway. J Biol Chem. 279(47):49523–49532. 15347683 10.1074/jbc.M402034200

[CIT0048] Li C, Zhao R, Gao K, Wei Z, Yin MY, Lau LT, Chui D, Yu AC. 2011. Astrocytes: implications for neuroinflammatory pathogenesis of Alzheimer’s disease. Curr Alzheimer Res. 8(1):67–80. 21143158 10.2174/156720511794604543

[CIT0049] Li R, Yang L, Lindholm K, Konishi Y, Yue X, Hampel H, Zhang D, Shen Y. 2004. Tumor necrosis factor death receptor signaling cascade is required for amyloid-β protein-induced neuron death. J Neurosci. 24(7):1760–1771. 14973251 10.1523/JNEUROSCI.4580-03.2004 PMC6730458

[CIT0050] Li Y, Liu L, Kang J, Sheng JG, Barger SW, Mrak RE, Griffin WS. 2000. Neuronal-glial interactions mediated by interleukin-1 enhance neuronal acetylcholinesterase activity and mRNA expression. J Neurosci. 20(1):149–155. 10627591 10.1523/JNEUROSCI.20-01-00149.2000 PMC6774108

[CIT0051] Liu CY, Wang X, Liu C, Zhang HL. 2019. Pharmacological targeting of microglial activation: new therapeutic approach. Front Cell Neurosci. 13(11):1–19. 31803024 10.3389/fncel.2019.00514 PMC6877505

[CIT0052] Liu Q, Liu JP, Mei JH, Li SJ, Shi LQ, Lin ZH, Xie BY, Sun WG, Wang ZY, Yang XL, et al. 2020. Betulin isolated from *Pyrola incarnata* Fisch. inhibited lipopolysaccharide (LPS)-induced neuroinflammation with the guidance of computer-aided drug design. Bioorg Med Chem Lett. 30(12):127193. 32334913 10.1016/j.bmcl.2020.127193

[CIT0053] Livak KJ, Schmittgen TD. 2001. Analysis of relative gene expression data using real-time quantitative PCR and the 2(-Delta Delta C(T)) method. Methods. 25(4):402–408. 11846609 10.1006/meth.2001.1262

[CIT0054] Maddula K, Kumar VP, Anusha JR. 2017. Assessment of aqueous extract of *Ocimum sanctum* leaves in memory enhancement and preventing memory impairment activities in Zebra Fish model. J Basic Clin Pharm. 8:185–192.

[CIT0058] Millycent S, Mwonjoria M, Juma K, Ngugi M, Njagi E. 2017. Evaluation of analgesic, anti-inflammatory and toxic effects of *Lantana camara* L. Int J Phytopharm. 8:89–97.

[CIT0059] Mohs RC, Schmeidler J, Aryan M. 2000. Longitudinal studies of cognitive, functional and behavioral change in patients with Alzheimer’s disease. Statist Med. 19(11-12):1401–1409. 10.1002/(sici)1097-0258(20000615/30)19:11/12<1401::aid-sim432>3.0.co;2-x 10844705

[CIT0060] Muller-Ebeling C, Ratsch C. 1989. Heilpflanzen der Seychellen: Ein Beitrag zur kreolischen Volksheilkunde. 1st ed. Berlin: VWB-Verlag fur Wissenschaft und Bildung, German

[CIT0061] National Research Council (US) 2011. Guide for the care and use of laboratory animals eighth Edition. p. 8 ed. Washington, DC: The National Academies Press.

[CIT0062] Nazario LR, Antonioli R, Jr, Capiotti KM, Hallak JE, Zuardi AW, Crippa JA, Bonan CD, da Silva RS. 2015. Caffeine protects against memory loss induced by high and non-anxiolytic dose of cannabidiol in adult zebrafish (*Danio rerio*). Pharmacol Biochem Behav. 135:210–216. 26099242 10.1016/j.pbb.2015.06.008

[CIT0063] Nilsson P, Loganathan K, Sekiguchi M, Matsuba Y, Hui K, Tsubuki S, Tanaka M, Iwata N, Saito T, Saido TC. 2013. Aβ secretion and plaque formation depend on autophagy. Cell Rep. 5(1):61–69. 24095740 10.1016/j.celrep.2013.08.042

[CIT0064] Nour A, Khan M, Sulaiman PI, Nour A. 2014. *In vitro* anti-acetylcholinesterase and antioxidant activity of selected Malaysian plants. Asian J Pharm Clin Res. 7:93–97.

[CIT0065] Nunes-Tavares N, Santos L, Stutz B, Brito-Moreira J, Klein W, Ferreira S, Mello F. 2012. Inhibition of choline acetyltransferase as a mechanism for cholinergic dysfunction induced by amyloid-peptide oligomers. J Biol Chem. 287(23):19377–19385. 22505713 10.1074/jbc.M111.321448 PMC3365976

[CIT0066] Parada-Turska J, Turski WA. 1990. Excitatory amino acid antagonists and memory: effect of drugs acting at N-methyl-D-aspartate receptors in learning and memory tasks. Neuropharmacology. 29(12):1111–1116. 2149871 10.1016/0028-3908(90)90034-o

[CIT0067] Pellow S, Chopin P, File SE, Briley M. 1985. Validation of open: closed arm entries in an elevated plus-maze as a measure of anxiety in the rat. J Neurosci Methods. 14(3):149–167. 2864480 10.1016/0165-0270(85)90031-7

[CIT0068] Perry RT, Collins JS, Wiener H, Acton R, Go RC. 2001. The role of TNF and its receptors in Alzheimer’s disease. Neurobiol Aging. 22(6):873–883. 11754994 10.1016/s0197-4580(01)00291-3

[CIT0069] Prieur EAK, Jadavji NM. 2019. Assessing spatial working memory using the spontaneous alternation Y-maze test in aged male mice. Bio Protoc. 9(3):e3162. 10.21769/BioProtoc.3162 PMC7854095 33654968

[CIT0070] Qiu Z, Gruol DL. 2003. Interleukin-6, beta-amyloid peptide and NMDA interactions in rat cortical neurons. J Neuroimmunol. 139(1-2):51–57. 12799020 10.1016/s0165-5728(03)00158-9

[CIT0071] Quintanilla RA, Orellana DI, González-Billault C, Maccioni RB. 2004. Interleukin-6 induces Alzheimer-type phosphorylation of tau protein by deregulating the cdk5/p35 pathway. Exp Cell Res. 295(1):245–257. 15051507 10.1016/j.yexcr.2004.01.002

[CIT0072] Ross EL, Weinberg MS, Arnold SE. 2022. Cost-effectiveness of aducanumab and donanemab for early Alzheimer disease in the US. JAMA Neurol. 79(5):478–487. 35344024 10.1001/jamaneurol.2022.0315 PMC8961406

[CIT0073] Roy A. 2018. Role of medicinal plants against Alzheimer’s disease. Int J Complement Alt Med. 11:205–208.

[CIT0074] Sambeth A, Riedel WJ, Smits LT, Blokland A. 2007. Cholinergic drugs affect novel object recognition in rats: relation with hippocampal EEG? Eur J Pharmacol. 572(2-3):151–159. 17659275 10.1016/j.ejphar.2007.06.018

[CIT0075] Saraf MK, Prabhakar S, Khanduja KL, Anand A. 2011. *Bacopa monniera* attenuates scopolamine-induced impairment of spatial memory in mice. Evid Based Complement Alternat Med. 2011:236186. 21607013 10.1093/ecam/neq038 PMC3095476

[CIT0076] Sarter M, Bruno JP, Givens B. 2003. Attentional functions of cortical cholinergic inputs: what does it mean for learning and memory? Neurobiol Learn Mem. 80(3):245–256. 14521867 10.1016/s1074-7427(03)00070-4

[CIT0077] Schliebs R, Arendt T. 2011. The cholinergic system in aging and neuronal degeneration. Behav Brain Res. 221(2):555–563. 21145918 10.1016/j.bbr.2010.11.058

[CIT0078] Serrano-Pozo A, Frosch MP, Masliah E, Hyman BT. 2011. Neuropathological alterations in Alzheimer disease. Cold Spring Harb Perspect Med. 1(1):a006189. 22229116 10.1101/cshperspect.a006189 PMC3234452

[CIT0079] Shaftel SS, Griffin WS, O'Banion MK. 2008. The role of interleukin-1 in neuroinflammation and Alzheimer disease: an evolving perspective. J Neuroinflammation. 5:7. 18302763 10.1186/1742-2094-5-7 PMC2335091

[CIT0080] Sinha P, Barocas JA. 2022. Cost-effectiveness of aducanumab to prevent Alzheimer’s disease progression at current list price. Alzheimers Dement. 8(1):e12256. 10.1002/trc2.12256 PMC8900580 35282659

[CIT0081] Sierra JA, Gilchrist K, Tabares-Guevara JH, Betancur-Galvis L, Ramirez-Pineda JR, González-Cardenete MA. 2022. Semisynthetic abietic and dehydroabietic acid derivatives and triptoquinone epimers interfere with LPS-triggered activation of dendritic cells. Molecules. 27(19):6684. 36235219 10.3390/molecules27196684 PMC9571164

[CIT0082] Stancampiano R, Cocco S, Cugusi C, Sarais L, Fadda F. 1999. Serotonin and acetylcholine release response in the rat hippocampus during a spatial memory task. Neuroscience. 89(4):1135–1143. 10362301 10.1016/s0306-4522(98)00397-2

[CIT0083] Suganthy N, Malar DS, Devi KP. 2016. *Rhizophora mucronata* attenuates beta-amyloid induced cognitive dysfunction, oxidative stress and cholinergic deficit in Alzheimer’s disease animal model. Metab Brain Dis. 31(4):937–949. 27188290 10.1007/s11011-016-9831-0

[CIT0084] Sun Y, Lai MS, Lu CJ, Chen R-C. 2008. How long can patients with mild or moderate Alzheimer’s dementia maintain both the cognition and the therapy of cholinesterase inhibitors: a national population-based study. Eur J Neurol. 15(3):278–283. 18290848 10.1111/j.1468-1331.2007.02049.x

[CIT0085] Suthar SK, Lee HB, Sharma M. 2014. The synthesis of non-steroidal anti-inflammatory drug (NSAID)–lantadene prodrugs as novel lung adenocarcinoma inhibitors via the inhibition of cyclooxygenase-2 (COX-2), cyclin D1 and TNF-α-induced NF-κB activation. RSC Adv. 4(37):19283–19293.

[CIT0086] Torres-Acosta N, O'Keefe JH, O'Keefe EL, Isaacson R, Small G. 2020. Therapeutic potential of TNF-α inhibition for Alzheimer’s disease prevention. J Alzheimers Dis. 78(2):619–626. 33016914 10.3233/JAD-200711 PMC7739965

[CIT0087] Varfolomeev EE, Ashkenazi A. 2004. Tumor necrosis factor: an apoptosis JuNKie? Cell. 116(4):491–497. 14980217 10.1016/s0092-8674(04)00166-7

[CIT0088] Vinutha B, Prashanth D, Salma K, Sreeja SL, Pratiti D, Padmaja R, Radhika S, Amit A, Venkateshwarlu K, Deepak M. 2007. Screening of selected Indian medicinal plants for acetylcholinesterase inhibitory activity. J Ethnopharmacol. 109(2):359–363. 16950584 10.1016/j.jep.2006.06.014

[CIT0089] Wang H, Kulas JA, Wang C, Holtzman DM, Ferris HA, Hansen SB. 2021. Regulation of beta-amyloid production in neurons by astrocyte-derived cholesterol. Proc Natl Acad Sci USA. 118(33):e2102191118. 34385305 10.1073/pnas.2102191118 PMC8379952

[CIT0091] Whitehouse PJ, Price DL, Clark AW, Coyle JT, DeLong MR. 1981. Alzheimer disease: evidence for selective loss of cholinergic neurons in the nucleus basalis. Ann Neurol. 10(2):122–126. 7283399 10.1002/ana.410100203

[CIT0092] Whitehouse PJ, Price DL, Struble RG, Clark AW, Coyle JT, Delon MR. 1982. Alzheimer’s disease and senile dementia: loss of neurons in the basal forebrain. Science. 215(4537):1237–1239. 7058341 10.1126/science.7058341

[CIT0093] WHO. 2021. Dementia. [updated 2 September 2021; accessed 2022 September 16, 2022]. https://www.who.int/news-room/fact-sheets/detail/dementia.

[CIT0095] Yadang FSA, Nguezeye Y, Kom CW, Betote PHD, Mamat A, Tchokouaha LRY, Taiwé GS, Agbor GA, Bum EN. 2020. Scopolamine-induced memory impairment in mice: neuroprotective effects of *Carissa edulis* (Forssk.) Valh (Apocynaceae) aqueous extract. Int J Alzheimers Dis. 2020:6372059. 32934845 10.1155/2020/6372059 PMC7479457

[CIT0096] Yermakova AV, O'Banion MK. 2001. Downregulation of neuronal cyclooxygenase-2 expression in end stage Alzheimer’s disease. Neurobiol Aging. 22(6):823–836. 11754989 10.1016/s0197-4580(01)00303-7

[CIT0097] Zanandrea R, Abreu MS, Piato A, Barcellos LJG, Giacomini A. 2018. Lithium prevents scopolamine-induced memory impairment in zebrafish. Neurosci Lett. 664:34–37. 29126775 10.1016/j.neulet.2017.11.010

[CIT0098] Zhang B, Gaiteri C, Bodea L-G, Wang Z, McElwee J, Podtelezhnikov AA, Zhang C, Xie T, Tran L, Dobrin R, et al. 2013. Integrated systems approach identifies genetic nodes and networks in late-onset Alzheimer’s disease. Cell. 153(3):707–720. 23622250 10.1016/j.cell.2013.03.030 PMC3677161

[CIT0099] Zhang H, Wei W, Zhao M, Ma L, Jiang X, Pei H, Cao Y, Li H. 2021. Interaction between Aβ and tau in the pathogenesis of Alzheimer’s disease. Int J Biol Sci. 17(9):2181–2192. 34239348 10.7150/ijbs.57078 PMC8241728

